# Habitat Selection and Risk of Predation: Re-colonization by Lynx had Limited Impact on Habitat Selection by Roe Deer

**DOI:** 10.1371/journal.pone.0075469

**Published:** 2013-09-19

**Authors:** Gustaf Samelius, Henrik Andrén, Petter Kjellander, Olof Liberg

**Affiliations:** Department of Ecology, Swedish University of Agricultural Sciences, Riddarhyttan, Sweden; Bangor University, United Kingdom

## Abstract

Risk of predation is an evolutionary force that affects behaviors of virtually all animals. In this study, we examined how habitat selection by roe deer was affected by risk of predation by Eurasian lynx – the main predator of roe deer in Scandinavia. Specifically, we compared how habitat selection by roe deer varied (1) before and after lynx re-established in the study area and (2) in relation to habitat-specific risk of predation by lynx. All analyses were conducted at the spatial and temporal scales of home ranges and seasons. We did not find any evidence that roe deer avoided habitats in which the risk of predation by lynx was greatest and information-theoretic model selection showed that re-colonization by lynx had limited impact on habitat selection by roe deer despite lynx predation causing 65% of known mortalities after lynx re-colonized the area. Instead we found that habitat selection decreased when habitat availability increased for 2 of 5 habitat types (a pattern referred to as functional response in habitat selection). Limited impact of re-colonization by lynx on habitat selection by roe deer in this study differs from elk in North America altering both daily and seasonal patterns in habitat selection at the spatial scales of habitat patches and home ranges when wolves were reintroduced to Yellowstone National Park. Our study thus provides further evidence of the complexity by which animals respond to risk of predation and suggest that it may vary between ecosystems and predator-prey constellations.

## Introduction

Risk of predation is an evolutionary force that affects behaviors of virtually all animals [1]. Behaviors to avoid predation often result from a compromise between maximizing energetic intake and minimizing the risk of predation; such behaviors include increased vigilance, reduced foraging time, reduced movements, habitat shifts, and changes in group size (see Creel et al. [2] and references therein). Recent returns of large carnivores in Europe and North America have sparked considerable interest in how ungulates respond behaviorally to these predators and the risk that they pose (e.g. [2-4]). However, the majority of these studies have focused on systems where wolves ( *Canis* *lupus* ) were the main predators, whereas behavioral responses by ungulates to other predators (e.g. stalking and ambush predators) remain largely unknown (but see Hunter and Skinner [5] and Ratikainen et al. [6]).

Habitat selection is defined as the process by which an animal chooses among habitats and is measured by the use of a habitat relative to its availability [7,8]. Animals select habitats at spatial scales ranging from geographical (1^st^ order) to landscape (2^nd^ order), home range (3^rd^ order), and microsite (4^th^ order) [7]. Similarly, animals select habitats at temporal scales ranging from hours and days (short-term) to seasons and years (long-term) [2]. Moreover, habitat selection may also vary with habitat availability in that habitat selection often is stronger when a habitat is rare than when it is common [9,10] – a pattern referred to as functional response in habitat selection [11]. Habitat selection by ungulates is often dictated by factors such as risk of predation, forage distribution, competition, and individual variation (see Kittle et al. [12] and references therein). Risk of predation has strong impact on habitat selection by elk ( *Cervus* *canadensis* ) in North America where recent reintroduction of wolves in Yellowstone National Park has had considerable impact on both daily and seasonal patterns in habitat selection by elk [2,13]. However, the influence of risk of predation on habitat selection by ungulates in other ecosystems and different predators is poorly known (but see Ratikainen et al. [6] for daily patterns in habitat selection of roe deer *Capreolus* *capreolus* ).

Roe deer are small-sized ungulates (adult body mass is 20-30 kg) that are common throughout most of Europe [14]. They have a high metabolic rate and require frequent intake of food which, in turn, results in relatively high activity throughout the day, although dawn and dusk are periods of peak activity [15]. The spatial organization of roe deer varies with sex and season; male roe deer defend intra-sexual territories in summer, whereas female roe deer are non-territorial throughout the year [16,17]. Moreover, the spatial dynamic of roe deer is density dependent in that home range sizes decrease as roe deer population density increases [18]. Eurasian lynx ( *Lynx* *lynx* , lynx hereafter) are the main predators of roe deer in Scandinavia [19,20] although red foxes ( *Vulpes* *vulpes* ) can be important predators of fawns during the first two months of life [21,22].

The objective of this study was to examine how habitat selection by roe deer was influenced by the risk of predation by lynx. Specifically, we compared how habitat selection by roe deer varied (1) before and after lynx re-established in the study area and (2) in relation to habitat-specific risk of predation by lynx. All analyses were conducted within home ranges (i.e. at the 3^rd^ order of selection as defined by Johnson [7]) and at the temporal scale of seasons. We predicted that roe deer would (1) alter their patterns of habitat selection when lynx re-colonized the study area and (2) avoid habitats in which the risk of lynx predation was greatest in line with recent findings that reintroduction of wolves in Yellowstone National Park had considerable impact on both daily and seasonal patterns in habitat selection by elk [2,13].

## Materials and Methods

### Ethics statement

Capture and handling procedures were approved by the Swedish Animal Welfare Agency and adhered to legal and ethical requirements for research on wild animals in Sweden (see below for collection and sampling methods). Access to the study area was granted by the Swedish National Forest Enterprise (Sveaskog). This study was not conducted in areas or involve activities for which we did not have permission, was not conducted on private land, and was not conducted on endangered or protected species.

### Study area

This study was conducted at Grimsö Wildlife Research Area (59° 60 N, 15° 16 E) in south-central Sweden from September 1984 to May 2007. Grimsö Wildlife Research Area (13,700 ha) is located in the southern boreal zone and is dominated by forests (73% cover) and large bogs (19% cover) – remaining areas include lakes and rivers (6% cover) and agricultural lands (2% cover). The forest at Grimsö Wildlife Research Area is owned by the Swedish National Forest Enterprise and is managed intensively for timber and pulp. It consists of a mosaic of fairly even-aged stands of various ages that have a rotation period of about 100 years. Main tree species are Scots pine ( *Pinus* *sylvestris* ) and Norway spruce (*Picea abies*), which are mixed with deciduous species such as birch ( *Betula* *pendula* and *B* *. pubescens* ), aspen ( *Populus* *tremula* ), rowan ( *Sorbus* *aucuparia* ), and willow ( *Salix* spp.). The understory is dominated by bilberry ( *Vaccinium* *myrtillus* ), lingonberry ( *Vaccinium* *vitis-idaea* ), heather ( *Calluna* *vulgaris* ), and common hair grass ( *Deschampsia* *flexuosa* ). Daily mean temperature ranges from 20°C in summer to -10°C in winter. The ground is generally snow-covered from December to March.

In addition to roe deer, other herbivores at Grimsö Wildlife Research Area included moose ( *Alces* *alces* ), mountain hares ( *Lepus* *timidus* ), brown hares ( *Lepus* *europaeus* ), and various small rodents (Muridae and Cricetidae spp.). Lynx re-colonized the study area naturally in 1995-1996 (the first known reproduction occurred in the summer of 1996) after having been absent from the area for > 30 years; the re-colonization by lynx went from virtual absence to > 1 lynx/100 km^2^ within 1 year [23]. There were 29 radio-marked lynx of which 16 were adults that used the Grimsö Wildlife Research Area during one or more years from 1997 to 2007 (Andrén, unpublished material). We define September 1984 to September 1994 as *before* re-colonization by lynx and September 1997 to May 2007 as *after* re-colonization by lynx. Roe deer population densities ranged about 3.6-10 and 1.1-6.2 roe deer/km^2^ before and after lynx re-colonized the study area, respectively, where roe deer population densities were estimated by pellet-group counts following Neff [24] and a defecation rate of 22 pellet-groups per day [25].

### Roe deer capture and telemetry

Roe deer were captured in wooden box-traps baited with standard livestock forage and animals were equipped with radio-collars weighing about 70-350 g (Televilt Int., Lindesberg, Sweden and Telonics Inc., Mesa, Arizona, *n* = 311 animals radio-collared in this study, but see below for restrictions on number of animals included in analyses). All animals included in this study were of known age as they were marked as fawns (although not radio-marked at this stage). Radio-collars were equipped with mortality sensors, which allowed us to investigate mortalities in the field and thereby determine (1) cause of death (where lynx predation was determined by puncture marks in the throat or from evidence in the snow) and (2) location of kill sites. Lynx are rarely successful in capturing roe deer when attacks are initiated from > 50 m away [19] and lynx-killed roe deer were rarely dragged > 20 m from kill sites (K. Sköld, Grimsö Wildlife Research Station, pers. comm.). The location where we encountered lynx-killed roe deer were thus good approximations of both the attack and kill site (see discussion of using distance-based versus classification-based analyses and their sensitivity to error below). Animals were located by triangulation (≥3 azimuths within 10 minutes) from a mini-bus equipped with a six-element Yagi antenna (Telonics TR-2, Telonics Inc.). Animals were located once to twice per week, although the schedule for locating animals varied somewhat throughout the study. Animals were located between 08:00 and 18:00 (but see animals monitored for diurnal patterns in habitat selection below). Telemetry error was estimated at < 150 m [26].

A sub-sample of roe deer was monitored throughout the 24-hour cycle to examine diurnal patterns in habitat selection (n = 11 animals in Jan and Feb 1994, 12 animals in May and June 1994, and 15 animals in May and June 1999 – but see below for limiting analyses to animals that had access to > 5% of all habitats except agricultural lands to ensure that animals were exposed to the same habitats). Animals were located every 12 hours with the tracking schedule shifted by 1 hour per day to achieve an even distribution throughout the 24-hour cycle.

### Habitat delineation

We used a digital map developed by the forestry company Sveaskog to prepare a habitat map in ArcGIS 9.1 (Environmental Systems Research Institute, Redlands, California). Forestry activities resulted in continuous turn-over of forest stands so we developed a habitat map for each year and season (see definitions of seasons below). We classified habitats as *clear cuts* (0-10 years after a cutting event), *young forests* (10-25 years after a cutting event), *middle-aged forests* (25-60 years after a cutting event), *old forests* (> 60 years after a cutting event), *bogs*, and *agricultural lands* (yards, pastures, and hay fields). Habitat classes followed those of Cederlund [27] except that we used 60 years instead of 80 years as the distinction between middle-aged and old forests. The transition from both (1) young forest to middle-aged forest and (2) middle-aged forest to old forest corresponded to that of mean age for pre-commercial and commercial thinning, respectively (although there was considerable variation in the age of stands at which these activities were performed). The size and proportion of clear cuts were similar before and after lynx re-colonized the study area (mean size_before_ = 7.0 ± 0.3 ha, mean size_after_ = 6.5 ± 0.2 ha, proportion of clear cuts_before_ = 8 ± 2%, proportion of clear cuts_after_ = 11 ± 1%). The mean size of habitat patches for all habitats combined was 5.4 ha (range = 0.1-300 ha).

### Seasons and age classes

We divided the year into two seasons based on the social and reproductive dynamics of roe deer following Kjellander et al. [18]: *spring and summer* (15 April -15 Sept for males and 15 May -15 Sept for females) and *fall and winter* (16 Sept -14 April for males and 16 Sept -14 May for females). We used 3 age classes following Loison et al. [28]; *subadults* (1-2 years old), *adults* (2-7 years old), and *old animals* (> 7 years old). We did not include fawns in our analyses because space use by fawns is not independent of their mothers during their first year of life [14].

### Habitat selection analyses

Error in telemetry data (e.g. misclassification of animal locations) can have large impact on habitat selection analyses [29-31]. The risk of such error and thus bias in habitat selection analyses increases in heterogeneous environments where habitat patches are small relative to telemetry error [29,31]. Eighty percent of the telemetry positions in our study had ≥3 of the 5 different habitat types within the telemetry error of 150 m (n = 2,001 telemetry positions included in the final analyses). We therefore used the Euclidean distance-based method by Conner et al. [30] to derive seasonal estimates of habitat selection for each individual because this method is less sensitive to telemetry error than are classification-based methods such as compositional analyses [30,31]. The distance-based method by Conner et al. [30] compares the mean distance between animal locations and each habitat (i.e. habitat use, *u*) with the mean distance between random points and each habitat (i.e. expected distances between animal locations and each habitat given the availability of habitats, *r*) for each animal and time period (in this case season). The ratio between these measures (called u/r-ratio hereafter) is used as a measure of habitat selection for each animal and time period [30]. This ratio is expected to equal 1.0 when there is no selection (i.e. *u* = *r*), < 1 when a habitat is preferred (i.e. *u* < *r*), and > 1 when a habitat is avoided (i.e. *u* > *r*).

We restricted our analyses on habitat selection to animals with ≥ 20 positions per season and we generated 30 random points per home range (see home range calculations below) by using the random number function in Hawth’s Analysis Tools (www.spatialecology.com) for ArcGIS 9.1 (an incremental area plot showed that there was only limited increase in the mean size of home ranges when the number of positions used for estimating home ranges was ≥ 20). Similarly, we restricted our analyses on habitat selection to animals that had access to > 5% clear cuts, young forests, middle-aged forests, old forests, and bogs to ensure that animals were exposed to the same habitats. We excluded (1) agricultural lands because of the low proportion of agricultural lands in the study area (mean access to agricultural lands was 0.8 ± 0.3% for animals included in this study) and (2) excursions (defined as use of a secondary area that was > 1.5 km away from the edge of the main cluster of positions where 1.5 km corresponds to 1.5 times the average diameter of home ranges in the study area as determined by Cederlund [27]) to avoid including areas that were only temporarily used (n = 7 excursions by 7 animals which corresponded to 0.98% of all positions). We calculated (1) distances between (A) animal locations and habitats and (B) random points and habitats by using the spatial join function in ArcGIS 9.1 where the habitat map was split by habitat to allow calculations and (2) habitat composition of home ranges (i.e. the proportion of habitats within home ranges) by using the intersect function in ArcGIS 9.1 where surface areas were recalculated by using the add area tool in Hawth’s Analysis Tools. We derived seasonal home ranges by calculating minimum convex polygons for each animal and season by using the minimum convex polygon function in Hawth’s Analysis Tools for ArcGIS 9.1.

### Habitat-specific risk of lynx predation

We developed a measure of habitat-specific risk of lynx predation by dividing the distance between each kill site and each habitat (k) with the mean distance between roe deer locations and each habitat (*u*), where the mean distance between roe deer locations and each habitat was based on all animals included in the study after lynx re-colonized the area and calculated separately for each sex and season (i.e. habitat-specific risk of predation corrected for habitat use, sex, and season). This ratio (called k/u-ratio hereafter) is expected to equal 1.0 when the risk of predation follows the rate at which a habitat is used (i.e. *k* = *u*), < 1 when the risk of lynx predation is high relative to the rate at which a habitat is used (i.e. *k* < *u*), and > 1 when the risk of lynx predation is low relative to the rate at which a habitat is used (i.e. *k* > *u*). The distance between kill sites and each habitat was calculated by using the spatial join function in ArcGIS 9.1 as described above.

### Statistical analyses

We examined how habitat selection by roe deer at the spatial and temporal scales of home ranges and seasons varied among habitats and in relation to re-colonization by lynx, age and sex of roe deer, season, roe deer population density, and habitat availability (i.e. the proportion of habitat available within home ranges) by a mixed linear model (Proc Mixed, SAS Institute Inc., Cary, North Carolina), where we controlled for repeated observation of individuals by using animal identity as random factor (*n* = 390 u/r-ratios from 52 individuals and 5 habitats). We derived 50 *a priori* candidate models where models ranged from none to all combinations of up to 2 of the independent variables above – including models both with and without two-way interactions between main effects (see Table S1 in Supporting Information for complete list of models). We used variation around the grand mean as a *null model* of no effect of either of the examined variables. We used Akaike’s information criterion (AIC) with small-sample adjustment (AIC_C_) to rank quality of models on habitat selection [32]. Further, we selected the model with the lowest AIC_C_ value as the best model and considered models within 2 AIC_C_ units to be of similar quality [32]. We examined how the risk of lynx predation varied among habitats by a mixed linear model (Proc Mixed, SAS Institute Inc.), where we controlled for repeated observation by using animal identity as a random factor (*n* = 195 k/u-ratios from 39 animals and 5 habitats). We compared the model of habitat-specific risk of lynx predation with a null model of no variation in the risk of lynx predation among habitats by using AIC_C_ described above.

We examined for potential bias in habitat selection analyses that may have been caused by the fact that the majority of data were collected between 08:00–18:00 by a repeated measures ANOVA (Proc ANOVA, SAS Institute Inc.) where we compared whether habitat selection by roe deer varied between day (08:00–18:00) and night (18:00–08:00). We performed analyses separately for each habitat and we performed analyses by controlling for season (*n* = 6 repeated measures for each comparison).

We present mean ± 95% CI unless otherwise stated.

## Results

The mean size of home ranges for female roe deer was 1.6 ± 0.5 in spring and summer and 1.8 ± 0.3 km^2^ in fall and winter, and the mean size of home ranges for male roe deer was 1.9 ± 0.4 in spring and summer and 1.5 ± 0.5 km^2^ in fall and winter. The average home range for roe deer was composed of 15 ± 1.7% clear cut, 15 ± 2.1% young forest, 28 ± 3.3% middle-aged forest, 21 ± 2.6% old forest, 20 ± 0.9% bogs, and 0.8 ± 0.3% agricultural land. Habitat selection by roe deer was similar for day and night (Table 1), although this analysis was limited by small sample size.

**Table 1:** Summary statistics for repeated ANOVAs examining whether habitat selection by roe deer at the spatial and temporal scales of home ranges and seasons differed between day (08:00–18:00) and night (18:00–08:00) at Grimsö Wildlife Research Area in 1994 and 1999.

**Habitat**	**u/r-ratio at day, mean ± 95% C.I.**	**u/r-ratio at night, mean ± 95% C.I.**	**F_1,4_**	***p***
Clear cut	1.18 ± 0.25	0.97 ± 0.58	4.62	0.098^a^
Young forest	1.02 ± 0.24	1.25 ± 0.52	1.45	0.725
Middle-aged forest	0.94 ± 0.39	0.97 ± 0.66	0.00	0.957
Old forest	1.07 ± 0.30	1.30 ± 0.49	0.96	0.383
Bog	0.97 ± 0.45	1.19 ± 0.29	3.34	0.142

^a^ the interaction between time of day and season (F_1,4_ = 9.21, *p* = 0.039) suggest that roe deer tended to avoid clear cuts at night in fall and winter although there was considerable overlap in confidence intervals of these estimates

**Figure 1:**
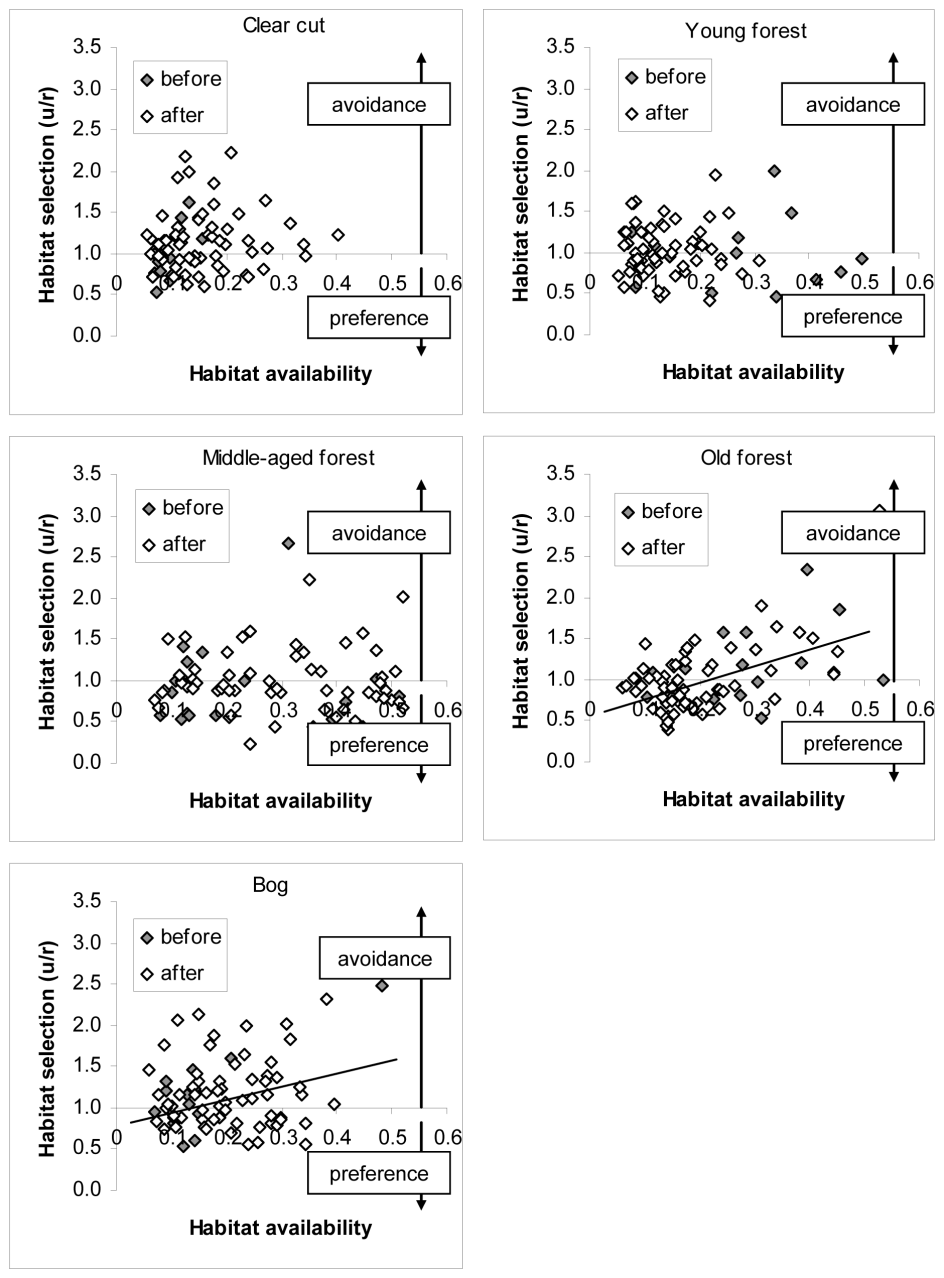
Habitat selection by roe deer at the spatial and temporal scales of home-ranges and seasons in relation to habitat availability at Grimsö Wildlife Research Area in 1984-2007. Trend-lines in figures illustrate the relationship between habitat selection and habitat availability for habitats in which this relationship was different from zero (the relationship between habitat selection and habitat availability was corrected for repeated observations of the same individuals). Random use is equal to one (i.e. a line following the x-axis).

The model {Habitat × Habitat Availability} described the variation in habitat selection at the spatial and temporal scales of home ranges and seasons better than other models and accounted for >99% of the model weight (Table 2). Specifically, habitat selection by roe deer at these scales varied between habitats and in relation to habitat availability in old forests and bogs, whereas the relationship between habitat selection and habitat availability at these scales was unclear for clear cuts, young forests, and middle-aged forests given that the slope estimates were not different from zero in these habitats (Figure 1); u/r-ratios increased by 0.21 ± 0.08 in old forests and 0.17 ± 0.12 in bogs when habitat availability increased by 10%, whereas the slope estimates were 0.07 ± 0.11, 0.004 ± 0.08, and -0.001 ± 0.07 for clear cuts, young forests, and middle-aged forests, respectively, when habitat availability increased by 10%. Re-colonization by lynx had limited impact on habitat selection by roe deer at the spatial and temporal scales of home ranges and seasons irrespective of age, sex, season, and roe deer population density, as illustrated by the models that included re-colonization by lynx (1) having 31-46 AIC_C_ units less support than the top model {Habitat × Habitat Availability} and (2) accounting for < 0.001% of the cumulative model weight (Table 2).

**Table 2:** Summary of top three models describing variation in habitat selection by roe deer at the spatial and temporal scales of home ranges and seasons at Grimsö Wildlife Research Area in 1984-2007 (see Table S1 for complete list of models).

**Model ^a^**	**K**	**Δ_i_**	***w*_i_**
Habitat × Habitat Availability	11	0	>0.99
Roe Deer Density × Habitat Availability	5	21.9	<0.001
Habitat × Roe Deer Density	11	24.0	<0.001
Null model (no variables included)	2	32.6	<0.001

Included in the table are differences in AIC_C_ values between each model and the best model (Δ_i_), number of model parameters (K), and model weights (*w*_i_). We used variation around the grand mean as our null model of no effect of either of the examined variables. Models that included re-colonization by lynx explained variation in habitat selection by roe deer 31-46 AIC_C_ units worse than the top model {Habitat × Habitat Availability} and accounted for <0.001% of the cumulative model weight.

a × indicates that both main effects and two-way interactions between main effects were included in the model

**Figure 2:**
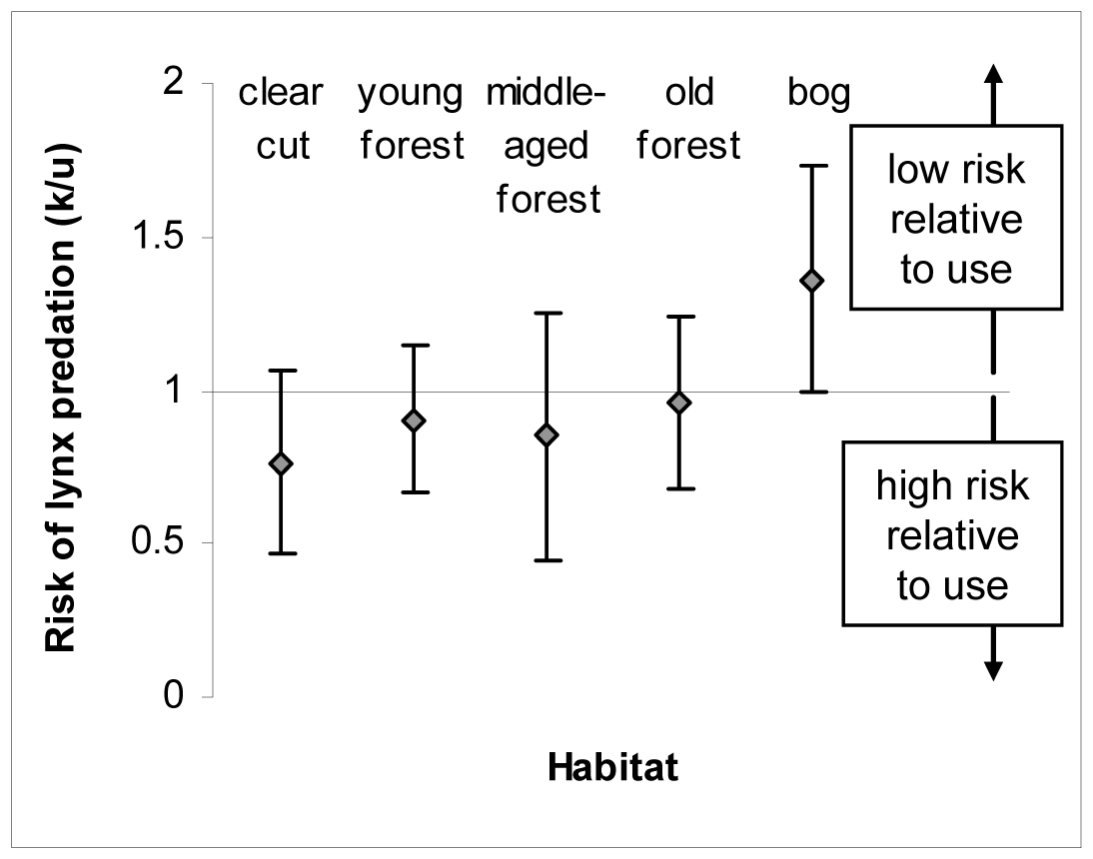
Habitat-specific risk of lynx predation (mean ± 95% CI) at the spatial and temporal scales of home-ranges and seasons at Grimsö Wildlife Research Area in 1997-2007. Neutral risk of predation is equal to one (i.e. a line following the x-axis).

Ninety-five radio-collared roe deer died during this study; lynx predation caused 65% of known mortalities after lynx re-colonized the study area and thus was the main cause of mortality during this period (*n* = 40 confirmed and 4 suspected lynx kills of 68 known mortalities after lynx re-established in the study area). The risk of lynx predation at the spatial and temporal scales of home ranges and seasons differed between habitats and was high on clear cuts, low on bogs, and intermediate in forest habitats as illustrated by the model that included habitat-specific risk of lynx predation (1) describing the variation in risk of predation 2.6 AIC_C_ units better than the null model of no variation in the risk of predation among habitats and (2) accounting for 79% of the model weight (Table 3, Figure 2).

**Table 3:** Estimates of habitat-specific risk of lynx predation at the spatial and temporal scales of home-ranges and seasons at Grimsö Wildlife Research Area in 1997-2007.

**Habitat**	**k/u-ratio, mean ± 95% C.I.**
Clear cut	0.77 ± 0.30
Young forest	0.90 ± 0.24
Middle-aged forest	0.85 ± 0.40
Old forest	0.96 ± 0.28
Bog	1.36 ± 0.37

## Discussion

How animals respond to risk of predation is a central issue in behavioral ecology [1]. This study showed that re-colonization by lynx had limited impact on habitat selection of roe deer at the spatial and temporal scales of home ranges and seasons despite lynx predation causing 65% of known mortalities after lynx re-colonized the study area. It is possible that re-colonization by lynx affected habitat selection by roe deer at finer scales than examined in this study, although roe deer in southern Norway selected winter habitats similarly at the spatial and temporal scales of habitat patches and days before and after lynx re-colonized the area [6]. Limited impact of re-colonization by lynx on habitat selection by roe deer on a range of scales in these studies differs from elk in North America altering both daily and seasonal patterns in habitat selection at the scales of habitat patches and home ranges when wolves were reintroduced in Yellowstone National Park [2,13]. Differences in how roe deer and elk responded to risk of predation in these studies may largely have been related to differences in hunting strategies by the predators or differences in foraging behaviors and social organization of the herbivores. For example, it may be more difficult to avoid predation by altering habitat selection when exposed to stalking predators that hunt alone (e.g. lynx) than when exposed to coursing predators that hunt in packs (e.g. wolves). Moreover, roe deer are small ungulates that require frequent intake of high-quality foods and therefore may be forced to move among habitats to track variation in the quality and abundance of plants and thereby also be forced to accept greater risk of predation than larger and less selective feeders such as elk (cf. Hofmann [33]). Similarly, the fact that roe deer in our study did not show any evidence of avoiding habitats in which the risk of lynx predation was greatest, further suggests that habitat selection by roe deer may be driven more strongly by foraging needs than by risk of predation (see Ratikainen et al. [6] for similar suggestion). Differences in how roe deer and elk responded to risk of predation may also be related to differences in social organization and vigilance, where roe deer live solitarily or in smaller groups than elk [14,34], and therefore may have a lower probability of detecting predators than elk. Similarly, the probability of surviving a predator-attack is generally greater in larger groups due to the dilution effect [34,35] and therefore it is possible that elk are more flexible in learning and adjusting their anti-predatory behaviors than are roe deer. Finally, differences in how roe deer and elk responded to risk of predation may also be related to differences in habitat composition and landscape structure, where our study and that of Ratikainen et al. [6] were conducted in forest landscapes managed for timber and pulp production, whereas the studies on habitat selection by elk in Yellowstone National Park were conducted in mountainous areas dominated by shrub and grassland [2,13].

This study showed that habitat selection by roe deer at the spatial and temporal scales of home ranges and seasons decreased when habitat availability increased in two of five habitats, which was similar to moose and red deer ( *Cervus* *elaphus* ) selecting less abundant habitats more strongly at a range of spatial and temporal scales [9,10]. There is thus evidence that habitat selection by ungulates is a dynamic process where ultimate strategies vary in relation to habitat availability and landscape composition. That habitat selection varies in relation to habitat availability may largely result from a trade-off in the time allocated for foraging versus resting and digesting of foods when the availability of habitats and resources vary in space and time as suggested by Mysterud and Ims [11]. Moreover, a flexible strategy in how to select and use space may be especially important for selective browsers such as roe deer that may be forced to move among habitats to track spatial and temporal variation in plant quality and abundance. A decrease in the relative use of abundant habitats may also be adaptive to reduce the risk of predation by preventing predators from learning the spatial distribution of their prey or focusing on commonly-used habitats in what is referred to as the game of confusion by Mitchell and Lima [36].

In summary, this study and that by Ratikainen et al. [6] showed that there are marked differences in how roe deer and elk respond to the risk of predation at both fine and large temporal and spatial scales which, in turn, suggest that the way animals respond to risk of predation vary among ecosystems and predator-prey constellations. Moreover, the lack of any marked effect of re-colonization by lynx on habitat selection by roe deer in our study and that by Ratikainen et al. [6] suggests that behaviorally-induced trophic cascades such as those observed when wolves were reintroduced in Yellowstone National Park (e.g. [2,37,38]) are unlikely to occur in roe deer-lynx systems.

## Supporting Information

Table S1 **Complete list of candidate models describing variation in habitat selection by roe deer at the spatial and temporal scales of home ranges and seasons at Grimsö Wildlife Research Area in 1984-2007.** Included in the table are differences in AIC_C_ values between each model and the best model (Δ_i_), number of model parameters (K), and model weights (*w*_i_). We used variation around the grand mean as our null model of no effect of either of the variables examined.
(DOC)
