# Supplementary material for: Habitat Selection and Risk of Predation: Re-colonization by Lynx had Limited Impact on Habitat Selection by Roe Deer
Source: PLoS One. 2013 Sep 19;8(9):e75469. doi: 10.1371/journal.pone.0075469 (PMC3777928; doi:10.1371/journal.pone.0075469)
Supplement: Table S1 — Complete list of candidate models describing variation in habitat selection by roe deer at the spatial and temporal scales of home ranges and seasons at Grimsö Wildlife Research Area in 1984-2007. Included in the table are differences in AICC values between each model and the best model (Δi), number of model parameters (K), and model weights (w i). We used variation around the grand mean as our null model of no effect of either of the variables examined. (DOC) [file pone.0075469.s001.doc]

| **Model ^a^** | **K** | **Δ_i_** | ***w*_i_** |
| --- | --- | --- | --- |
| Habitat × Habitat Availability | 11 | 0 | >0.99 |
| Roe Deer Density × Habitat Availability | 5 | 21.9 | <0.001 |
| Habitat × Roe Deer Density | 11 | 24.0 | <0.001 |
| Habitat + Habitat Availability | 7 | 24.0 | <0.001 |
| Age × Habitat Availability | 7 | 27.7 | <0.001 |
| Habitat Availability | 3 | 28.3 | <0.001 |
| Roe Deer Density + Habitat Availability | 4 | 29.1 | <0.001 |
| Lynx Colonization × Habitat Availability | 5 | 31.0 | <0.001 |
| Season × Habitat Availability | 5 | 31.9 | <0.001 |
| Season + Habitat Availability | 4 | 32.1 | <0.001 |
| Sex + Habitat Availability | 4 | 32.5 | <0.001 |
| Lynx Colonization + Habitat Availability | 4 | 32.5 | <0.001 |
| *null model (no variables included)* | 2 | 32.6 | <0.001 |
| Sex × Habitat Availability | 5 | 32.8 | <0.001 |
| Roe Deer Density | 3 | 33.4 | <0.001 |
| Age × Roe Deer Density | 7 | 34.4 | <0.001 |
| Lynx Colonization × Roe Deer Density | 5 | 35.2 | <0.001 |
| Age + Habitat Availability | 5 | 35.5 | <0.001 |
| Habitat | 6 | 35.7 | <0.001 |
| Sex × Roe Deer Density | 5 | 35.8 | <0.001 |
| Season × Roe Deer Density | 5 | 36.2 | <0.001 |
| Season | 3 | 36.4 | <0.001 |
| Habitat + Roe Deer Density | 7 | 36.5 | <0.001 |
| Sex | 3 | 36.8 | <0.001 |
| Lynx Colonization | 3 | 36.8 | <0.001 |
| Lynx Colonization + Roe Deer Density | 4 | 36.9 | <0.001 |
| Season + Roe Deer Density | 4 | 37.0 | <0.001 |
| Sex + Roe Deer Density | 4 | 37.6 | <0.001 |
| Habitat + Season | 7 | 39.6 | <0.001 |
| Age | 4 | 39.7 | <0.001 |
| Habitat + Lynx Colonization | 7 | 39.9 | <0.001 |
| Habitat + Sex | 7 | 40.0 | <0.001 |
| Age + Roe Deer Density | 5 | 40.5 | <0.001 |
| Season + Lynx Colonization | 4 | 40.6 | <0.001 |
| Sex + Season | 4 | 40.7 | <0.001 |
| Sex + Lynx Colonization | 4 | 41.0 | <0.001 |
| Sex × Lynx Colonization | 5 | 42.8 | <0.001 |
| Habitat + Age | 8 | 42.9 | <0.001 |
| Season × Lynx Colonization | 5 | 43.2 | <0.001 |
| Sex × Season | 5 | 43.3 | <0.001 |
| Age + Season | 5 | 43.3 | <0.001 |
| Age + Sex | 5 | 43.6 | <0.001 |
| Age + Lynx Colonization | 5 | 43.7 | <0.001 |
| Habitat × Season | 11 | 44.9 | <0.001 |
| Habitat × Lynx Colonization | 11 | 45.6 | <0.001 |
| Age × Lynx Colonization | 7 | 45.7 | <0.001 |
| Habitat × Sex | 11 | 45.8 | <0.001 |
| Age × Sex | 7 | 46.1 | <0.001 |
| Age × Season | 7 | 46.9 | <0.001 |
| Habitat × Age | 16 | 48.1 | <0.001 |

^a^ × indicates that both main effects and two-way interactions between main effects were included in the model whereas + indicates that only main effects were included in the model
